# Teleducation: medical education in the pandemic and beyond

**DOI:** 10.3389/fmed.2023.1251732

**Published:** 2023-11-07

**Authors:** Tse Kiat Soong, Lloyd Jee Hean Ng, Joel Wen Liang Lau, Choon Seng Chong, James Wai Kit Lee

**Affiliations:** ^1^Alexandra Hospital, National University Health Systems, Singapore, Singapore; ^2^Yong Loo Lin School of Medicine, National University of Singapore, Singapore, Singapore; ^3^National University Hospital, National University Health Systems, Singapore, Singapore; ^4^Ng Teng Fong General Hospital, National University Health Systems, Singapore, Singapore; ^5^Ark Surgical Practice, Mount Elizabeth Hospital, Singapore, Singapore

**Keywords:** telegram, medical education, pandemic, teaching, education technologies

## Abstract

Medical education in the pandemic has been challenging owing to various physical and technological constraints in the current education landscape. This has resulted in reduced patient contact and opportunities for clinical exposure. In utilizing various platforms to supplement teaching, we adopted the use of Telegram, a cloud-based messaging application as an education aid for 3 cohorts of medical students in 1 medical school in Singapore. Herein, we share our experience with Telegram as a novel platform to augment medical education and to supplement clinical training amidst the various constraints. We believe that the circumstances have allowed us to find a method that may serve as an effective adjunct in education. Qualitative feedback has been positive and generally in line with our goals. We believe that further work could involve utilizing other features of the application, or by developing specialized applications to serve the same purpose. More needs to be done to consider applicability in different cultural and socioeconomic contexts.

## 1. Introduction

The COVID-19 pandemic greatly affected clinical based medical education. Student-patient and student-doctor interactions which are an integral component of medical education have been limited due to restrictions put in place to curb the spread of COVID-19. This, undoubtedly, served as a source of concern for many medical students, citing the impact of markedly reduced clinical exposure (1). As a result, digital adaptation of technology using applications such as Zoom and/or Microsoft Teams have been used to instruct medical students while physical tutorials and lessons were minimized.

Instead of physical tutorials, Zoom-based tutorials have become the new-normal over the past year. However, attending multiple Zoom based lectures over the day have brought forth the issue of “Zoom fatigue.” This is attributed to non-verbal overload, namely: reduced mobility, mirror effect of looking at oneself on the screen, increased cognitive load of sending and reading non-verbal signals, and prolonged eye gaze at close distances (2).

Instead of delivering medical education *via* a constant stream of lectures, we decided to compartmentalize teaching into bite-sized content. We chose to utilize Telegram, a cloud-based messaging application which can create channels, polls, host unlimited file sharing content and to foster discussion. (3) Beyond the above capabilities, Telegram has access to various official and user-created bots which add further functionality to the platform, ranging from daily reminders for important events of the day to even checking the time for the next available bus at a given station.

In this article, we define our experience with the platform as a teaching tool and provide examples as to the methods of utilization of some functions within the application. Through piloting medical education *via* the Telegram application, we aimed to show that the platform was suitable to enhance student-tutor interaction, supplement learning for medical students, foster interactions among students and be a sustainable mode of education. We hypothesize that the adoption of teaching *via* the Telegram application has the potential to augment medical education *via* the above factors, with possible applications beyond the pandemic.

## 2. Pedagogical principles

Cognitivism (4) has served as a guiding pillar for much of medical education’s history, with the focus on lectures, tutorials and use of medical textbooks forming the backbone of acquiring medical knowledge. This method has its disadvantages, such as the lack of consideration for socio-cultural effects on cognitive development and the utility of interactive learning (5).

In our current era, medical education has progressed to involve problem-based learning, encompassing connectivist (6), constructivist (7), and humanist (8) approaches to optimize learning. (9, 10) It is neither fully apprenticeship-based nor classroom based. Discussions and problem-based learning help bridge the gap between theory and applied knowledge in clinical medicine.

Telegram was selected as a platform as it was the most used messaging application among students at our institution. Also, it has advanced features over other mobile chat messaging applications. Telegram’s features include comments for each broadcast message, allowing for organized forum-like discussions; quizzes for single-best answer questions; polls that allow feedback to be gathered. In our study, we mainly used the ability to create a forum styled Telegram group to facilitate questions by topic, file sharing for important information and summaries of various teachings, as well as polls to get feedback on the deficiencies that may need further coverage by the tutors. This allows for us to apply the principles of connectivism and constructivism to facilitate problem-based learning for our students, and in so doing, bridge the gap between theory and applied clinical medicine.

We attempted to achieve this by creating 3 forum-styled Telegram groups. This was to aid with segmenting each group for its specific purposes – for information and material sharing, for clarification of doubts, and for application of their knowledge with various clinical pictures and vignettes as cases. Polls were used on a 2-weekly basis for assessment of topics that the students felt that they were weaker in to clarify doubts. Cases and vignettes were given as threads within the Telegram to allow for various students to answer and discuss the various points given within the case.

## 3. Learning objectives, environment, and pedagogical formats

**Figure 1:**
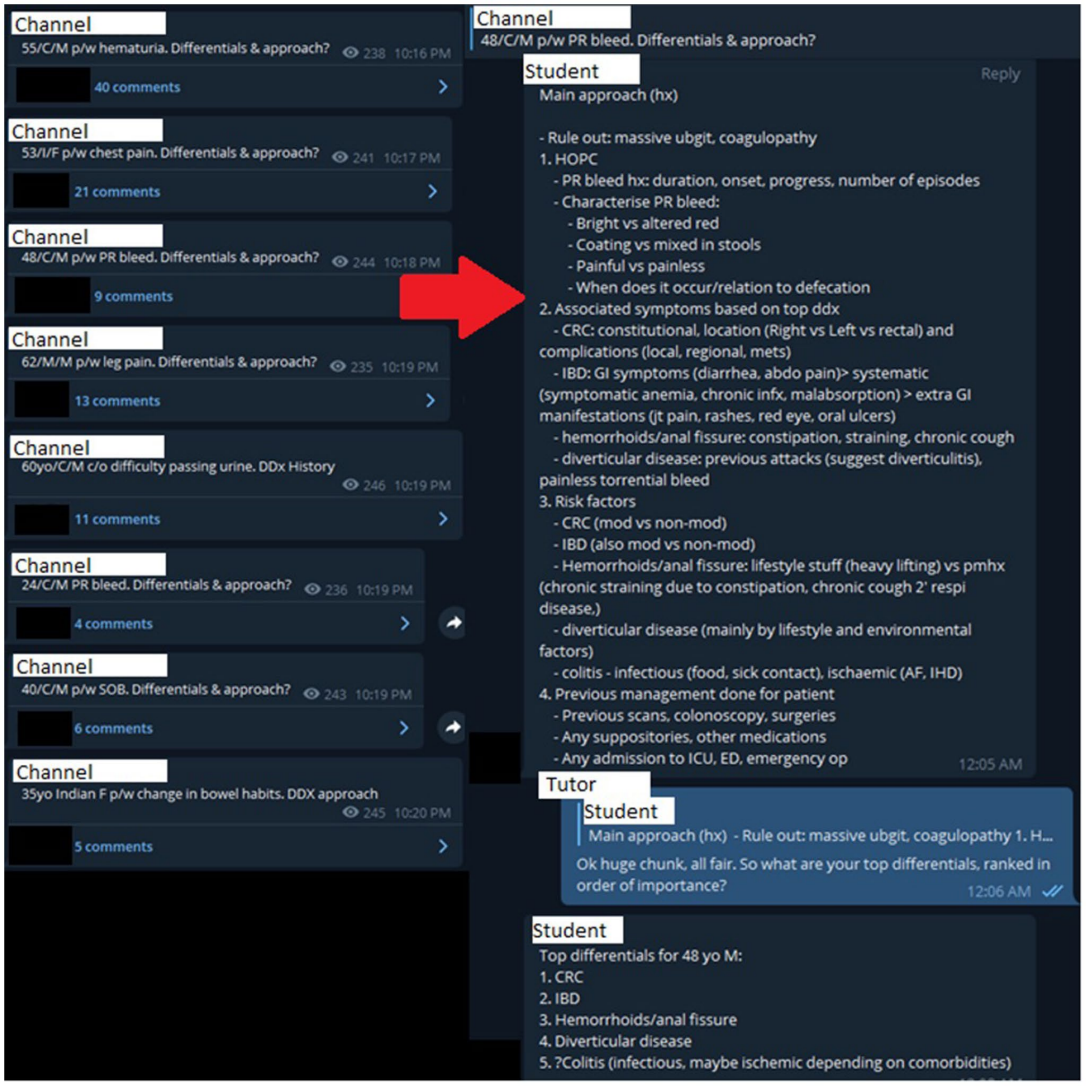
Case discussion on a 48 year-old Chinese male presenting with per-rectal bleeding. PR: Per-rectal; Hx: History; HOPC: History of presenting complaint; CRC: Colorectal cancer; IBD: Inflammatory bowel disease; GI: Gastrointestinal; AF: Atrial fibrillation; IHD: Ischaemic heart disease; ICU: Intensive care unit; ED: Emergency department.

We started to engage medical students in Singapore *via* the Telegram application from March 2020. We targeted undergraduate medical students on their General Surgery clinical rotations, from years 3 to 5. Each cohort has roughly 300 students. We utilized Telegram channels, a function which allows for “broadcasting” messages - or, in our case, clinical cases and questions (Figure 1). The forum-style channels allowed for students to leave comments on the main question, fostering discussion and allowing for medical educators to clarify doubts should they arise and progress the question in a sequential fashion as necessary. This was used as a tool to supplement the teaching of medical students. Using channels, students were exposed to common clinical scenarios and were challenged to formulate an appropriate clinical approach.

**Figure 2:**
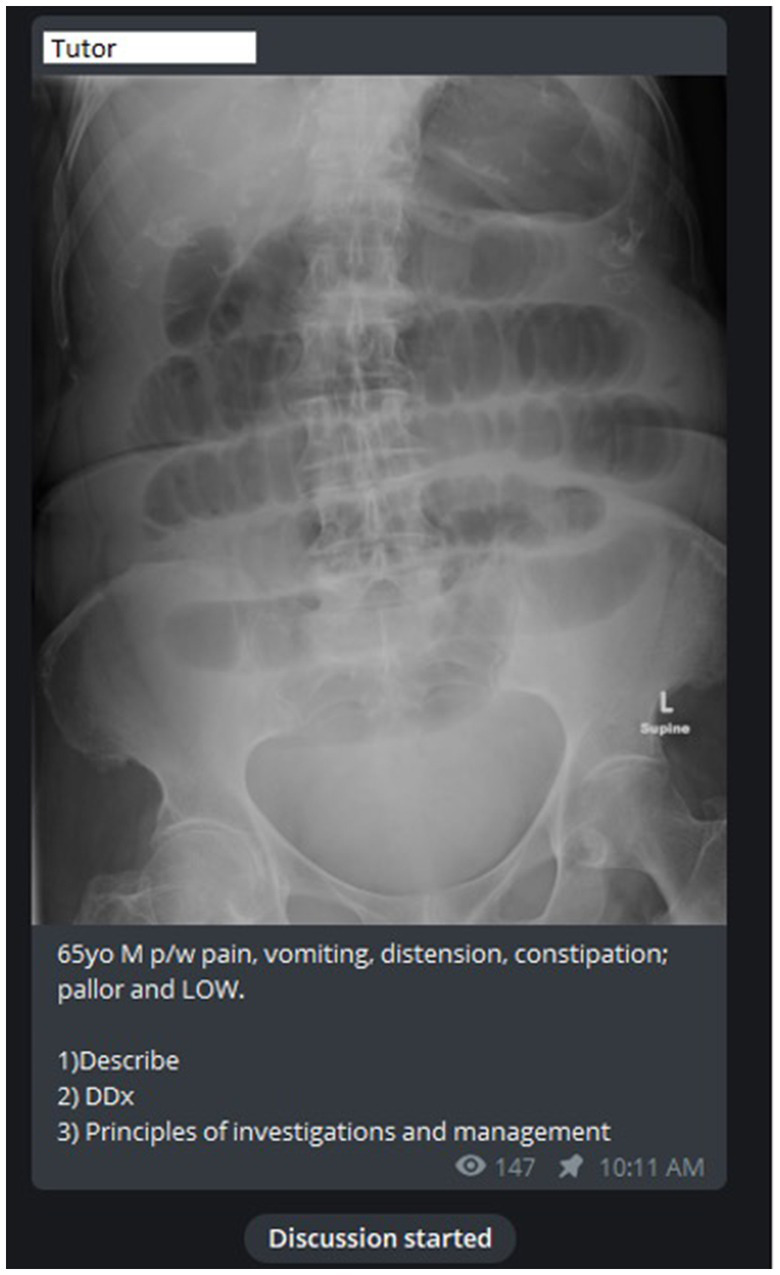
Case discussion with an abdominal radiograph of a 65 year old male. P/W: Presenting with; DDx: Differential diagnoses.

**Figure 3:**
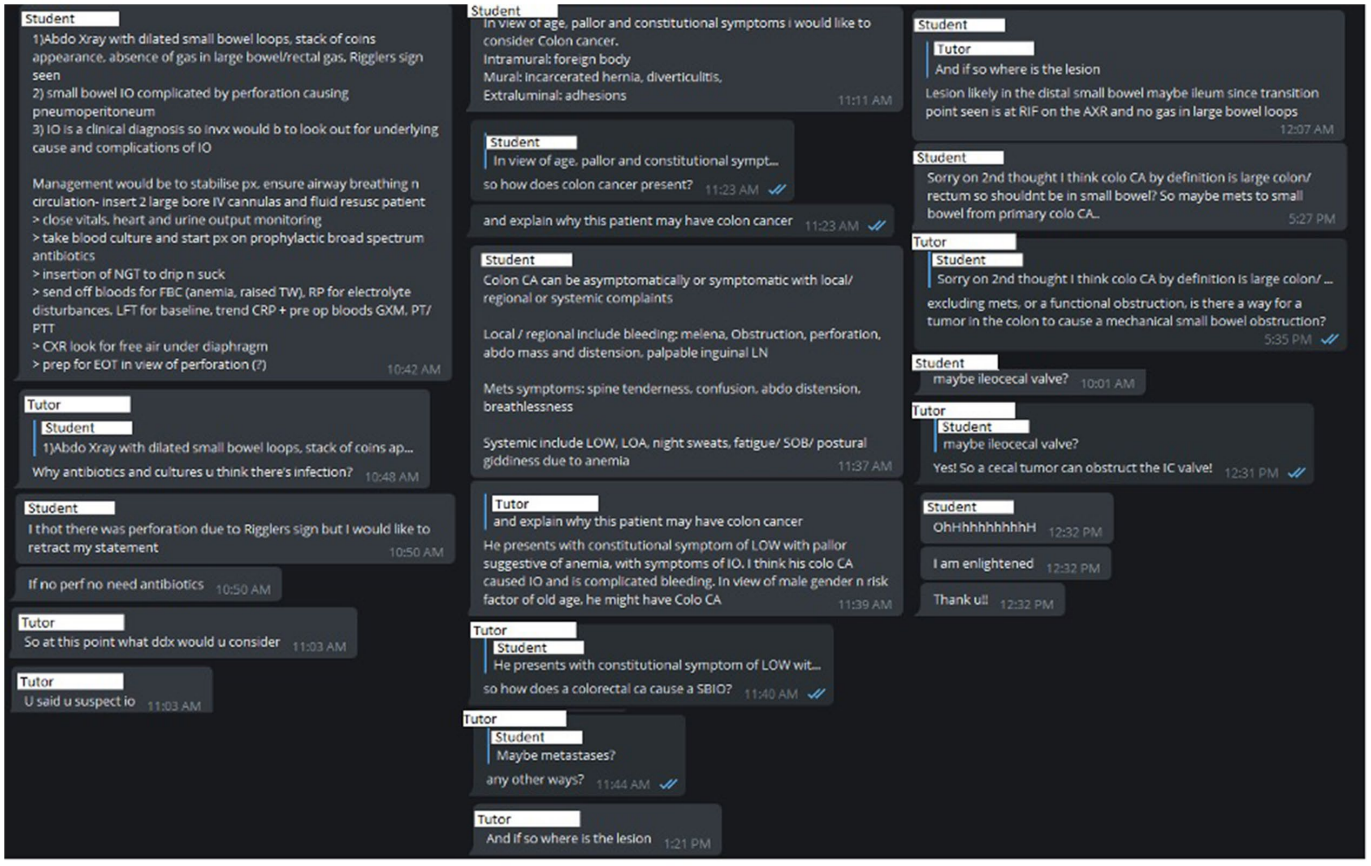
Discussion regarding case in Figure 2. IO: Intestinal obstruction; IV: Intravenous; NGT: Nasogastric tube; FBC: Full blood count; TW: Total white count; RP: Renal panel; LFT: Liver function tests; CRP: C Reactive Protein; GXM: Group and cross match; PT/PTT: Coagulation panel; CA: Cancer; LOW: Loss of weight; LOA: Loss of appetite; LN: Lymph nodes; SOB: Shortness of breath; SBIO: Small bowel intestinal obstruction; RIF: Right iliac fossa; AXR: Abdominal X-Ray; IC: Ileocecal.

The platform served to encourage greater application in clinical scenarios *via* the use of Objective Structured Slide Examination (OSSE) style questions to allow students to apply their knowledge to a clinical scenario (Figure 2). The platform allows for effective student-tutor interaction where immediate clarification of any misconception can be corrected in a targeted and timely fashion (Figure 3). Also, as multiple students can engage in the channel-based discussion, this allows for mutual learning.

The benefit of using the channels function also allowed for nomination of multiple administrators (i.e., teaching assistants) to assist the owner (i.e., consultant medical educator) in chairing the discussion sessions. This provided the opportunity for a larger pool of faculty to reach out to the same audience set, allowing for a greater diversity of discussion.

## 4. Assessment of interventions

We believe that we have largely met the goals we sought to achieve with this project.

We believe that the quality and quantity of discussions show that Telegram channels can serve as a suitable **platform for student-tutor interaction** (Figure 1). Organization of cases into separate threads, file sharing capabilities, reply functions, and polls to gather feedback provide the necessary functions required for effective interaction.

Additionally, the goal of **supplementing learning** was met. With 24/7 access, Telegram allows tutors and students to respond and prepare at their convenience. While clinical teaching should ideally take place during office hours, we note that most discussions took place after hours. Heavy clinical loads often force tutors to hold tutorials after-hours or be wrought with delays and cancelations. This platform eliminates logistical issues such as gathering and scheduling constraints and creates opportunities for students and tutors to interact at each other’s convenience. It offers an additional benefit of working across time zones. This adds another layer of potential in education-based international collaboration.

We hoped to have achieved more **inter-participant discussion**. Perhaps the lack of anonymity, and the inability to hide previous responses that may serve as “spoilers,” have resulted in largely one or two participants attempting each case. The authors believe that discussions between participants could allow them to learn from one another and rely less on tutor input.

The authors feel that Teleducation has also allowed more **efficient outreach** than conventional tutorials, since a single tutor could work with multiple students at once. We also believe that this is a sustainable method of education with potential - the platform allows for training of new tutors and educators that can train subsequent batches of participants. The hope is for participants to improve, and eventually lead discussions with fellow participants and juniors.

In terms of metrics of the platform, participation was about 82.0–90.3% for the students from Years 3–5 of the first batch, excluding the tutors. The average number of responses per thread ranged from 2–47, with a median response of 21 responses. Response times by tutors were prompt, as the threads were created often during hours when the tutors were available and during waking hours (up to about 11:00 PM), and when posts are scheduled, responses would be within the hour as there is a small team of tutors assisting for this endeavor.

For objective evaluation, we plan to administer pre- & post-application surveys to qualitatively define the effectiveness of the platform. The authors felt a head on comparison was unfair, since it would mean purposefully denying an outlet of education for those not in the intervention arm in these trying times. Additionally, these surveys were not previously administered for several reasons. The channel was originally born out of necessity, to help teach in a time where COVID-19 made clinical teaching and face-to-face discussions largely non-feasible. Much of it was rudimentary in nature and started as a small group as a trial. Only later did the idea fully evolve to its current state, with the channel starting in Mar 2020. Moreover, it was logistically difficult as it did not coincide with the academic year, causing it to be difficult to accurately gage the effectiveness of said interventions. Further evaluation will be performed in the coming academic year. Sample surveys are provided in the Supplementary Materials (Supplementary Material I & II). This will likely be implemented as part of the end of Surgery posting evaluation to assist in capturing all students who have been involved in the group to get their feedback and to serve as a touch point for any glaring issues that can be rectified as we continue to use the platform.

## 5. Discussions, constraints, and lessons learned

Using Telegram as an education platform is novel and has been rapidly adopted owing to limitations put forth by the pandemic (11). Its flexibility and functionality provide a robust springboard for educators. Also, it can help better equip our students with the thought processes and knowledge required to function as a doctor.

An added benefit is that the application allows for closer, guided interaction between tutors and students. Medical education *via* telegram forum style allows for assessment of answers and prompt clarification of any doubts or mistakes. Additionally, if there are any questions about the topic, students can leave a comment to further discuss the point, allowing everyone to benefit from their doubts immediately. This fosters interactive learning and is a useful adjunct to the traditional didactic style of education which most people are used to.

Having a discussion on a forum-style app allows for retrospective review of discussions by active and passive participants alike. This contrasts with face-to-face tutorials, where there is by default no documentation of a discussion.

Nonetheless, we concede that there exist various challenges. Telegram’s main purpose as a messaging platform can serve as a distraction, as raised by various other authors. “Some students found the application a source of disturbance and distraction while studying,” with feedback indicating that the constant notifications from groups and channels can be distracting (3). However, in our experience, this has been mitigated by the “always on” nature since students are free to answer at their leisure. Also, its scalability can also be viewed as a weakness. While there is the possibility of greater outreach with an economy of effort, this might skew the teacher-to-student ratio, which can reduce the effectiveness of using telegram as a tool to augment medical education. Building on this, the number of participants as well as its public nature might serve as deterrence toward active participation. While passive learning has its benefits such as better test scores, active learning yielded better understanding of the target concept.4 This delicate balance between active and passive learning and its benefits is an important issue to deliberate and achieve.

While we feel that Telegram has potential, we concede that much of what we have achieved is but a basic utilization of the platform, and that its use as a teaching aid is still in its infancy. Drawing from other sources, future directions can include bite-sized information messages like Journal Feed (12), which features updates in Emergency Medicine in short and succinct summaries. Additionally, new platforms with additional features could further facilitate learning and participation. Features could include summarization of new landmark studies or guidelines, allowing for discussion of such topics, and quiz/game styled based cases to allow gamification which may enhance learning. Also, anonymized replies can help to improve participation, and it is useful for students who are less confident in answering on a public domain. Prompts and guiding questions can also be supplied on demand for challenging questions. Similarly, an upvote tool could prove useful in incentivizing high-quality contributions to the discussions. These can also be helpful to passive reviewers by directing their attention to more helpful or popular answers.

To improve scalability, artificial intelligence may prove useful as automated answering bots. Ideally, these could prompt the participant for missing elements in their answers to encourage further critical thinking. In a more primitive form, automated bots could privately reveal suggested answers to participants after a response. This reduces the time needed to respond to individual answers.

The possibilities are endless, and these barely scrape the surface of what is possible. We hope that this could play a role in optimizing education resources and improving the quality of medical education, but also note that applicability can be limited by different cultural and socioeconomic practices. Nonetheless, we hope that this proof of concept might inspire the use of locally relevant resources to supplement education.

## 6. Conclusion

Teleducation is a useful tool that medical education institutions can consider adding to their arsenal of teaching pedagogies. It not only augments but provides a useful constant presence in an “always on” fashion to engage and push students to explore topics in more detail as well as further their interests in areas of medical science. However, much more can be done to improve content delivery and assess quantitatively and qualitatively the effect of this new novel teaching pedagogy in undergraduate medical education.

## Data availability statement

The original contributions presented in the study are included in the article/supplementary material, further inquiries can be directed to the corresponding author.

## Author contributions

All authors contributed to the conceptualization, manuscript preparation, editing and proofreading. All authors contributed to the article and approved the submitted version.
