# Supplementary material for: Teleducation: medical education in the pandemic and beyond
Source: Front Med (Lausanne). 2023 Nov 7;10:1251732. doi: 10.3389/fmed.2023.1251732 (PMC10662128; doi:10.3389/fmed.2023.1251732)
Supplement: Supplementary file 1 [file Data_Sheet_1.docx]

Supplementary Material

Teleducation: Medical Education in the Pandemic & Beyond

Soong Tse Kiat^1,4^, Lloyd Ng Jee Hean^2,4^, Joel Lau Wen Liang^3,4^, Chong Choon Seng^4,5^, James Lee Wai Kit^1,4*^

^1^Alexandra Hospital, National University Health Systems, Singapore

^2^National University Hospital, National University Health Systems, Singapore

^3^Ng Teng Fong General Hospital, National University Health Systems, Singapore

^4^National University of Singapore, Singapore

^5^Ark Surgical Practice, Mount Elizabeth Hospital, Singapore

*** Correspondence:**Dr James Lee Wai Kit
[james_lee@nuhs.edu.sg](mailto:james_lee@nuhs.edu.sg)

Supplementary Appendix I: Pre-survey for evaluation of the Telegram Channel

| **Question** | | **Format of question** |
| --- | --- | --- |
| **Demographics** | | |
| 1 | Which medical school are you from? | Select one option: Yong Loo Lin School of Medicine  Lee Kong Chian School of Medicine  Duke-NUS Medical School  International - Australia  International - US  International - UK  International - Ireland  Others: Please specify |
| 2 | Which year of study are you in? | Select one option:  Phase/Year/MS I  Phase/Year/MS II  Phase/Year/MS III  Phase/Year/MS IV  Phase/Year/MS V  Phase/Year/MS VI |
| 3 | What is your gender? | Select one option:  Male  Female  Others |
| 4 | What is your age? | Select one option:  18 - 21  22 - 24  25 and above |
| 5 | Do you currently reside in Singapore? | Select one option: Yes  No |
| 6 | Which years of study do you get your General Surgery rotations in your school? | Select multiple: Phase I  Phase II  Phase III  Phase IV  Phase V  Phase VI |
| **Assessment** | | |
|  | On a scale of 1 to 10, how much would you rate your understanding of General Surgery? | Numeric scale: 1-10 |
|  | Which subspecialties do you feel are your weakest points? | Select multiple:  Hepatobiliary  Colorectal  Endocrine  Head & Neck  Urology  Trauma/Critical Care  Breast  Vascular  Upper GI |
|  | How do you normally study for General Surgery? | Select multiple: Textbooks  Senior’s Notes  UpToDate  Online Websites  Clinics  Tutorials  Operating Theatre  Discussion with Friends, Seniors & Tutors |
|  | Which mediums do you find most useful for preparation & study of General Surgery? | Select multiple: Textbooks  Senior’s Notes  UpToDate  Online Websites  Clinics  Tutorials  Operating Theatre  Discussion with Friends, Seniors & Tutors |
|  | Has COVID-19 adversely affected your study of General Surgery? | Choose one: Yes  No  Neutral |
|  | Please rate how much it has affected your study of General Surgery. | Choose one:  Very severe detriment  Severe detriment  Moderate detriment  Mild detriment  No effect  Mild improvement  Moderate improvement  Large improvement  Very large improvement |
|  | How have your study patterns for General Surgery changed after the pandemic? Please select the mediums by which you are using more during this pandemic. | Choose multiple: Textbooks  Senior’s Notes  UpToDate  Online Websites  Clinics  Tutorials  Operating Theatre  Discussion with Friends, Seniors & Tutors |
|  | How helpful do you feel a Telegram channel for discussion of approaches, OSSEs, and case discussions would assist in your study of General Surgery? | Numerical scale: 1-10 |
|  | What do you most often use Telegram for? | Leisure Work Education  Others |
|  | How interested are you in General Surgery as a career? | Numerical scale 1-10 |
|  | How interested are you in General Surgery as a HO/MO posting? | Numerical scale 1-10 |
| **Outreach** | | |
|  | Have you heard about the Teleducation initiative for General Surgery? | Select one: Yes  No |
|  | (Conditional) If so, how did you hear about it? | Select multiple: Word of mouth from peers  NUH GS Posting  Recommended by tutors  Recommended by seniors |

Supplementary Appendix II: Post-survey for the evaluation of the Telegram Channel

| **Question** | | **Format of question** |
| --- | --- | --- |
| **Demographics** | | |
| 1 | Which medical school are you from? | Select one option: Yong Loo Lin School of Medicine  Lee Kong Chian School of Medicine  Duke-NUS Medical School  International - Australia  International - US  International - UK  International - Ireland  Others: Please specify |
| 2 | Which year of study are you in? | Select one option:  Phase/Year/MS I  Phase/Year/MS II  Phase/Year/MS III  Phase/Year/MS IV  Phase/Year/MS V  Phase/Year/MS VI |
| 3 | What is your gender? | Select one option:  Male  Female  Others |
| 4 | What is your age? | Select one option:  18 - 21  22 - 24  25 and above |
| 5 | Do you currently reside in Singapore? | Select one option: Yes  No |
| 6 | Which years of study do you get your General Surgery rotations in your school? | Select multiple: Phase I  Phase II  Phase III  Phase IV  Phase V  Phase VI |
| 7 | Are you aware of the Telegram initiative for General Surgery? | Yes  No |
|  | (Conditional) If so, how did you hear about it? | Select multiple: Word of mouth from peers  NUH GS Posting  Recommended by tutors  Recommended by seniors |
| **Assessment** | | |
|  | How useful was the Telegram channel in assisting with learning General Surgery? | Not useful  Minimally useful  Neutral  Somewhat useful  Very useful |
|  | How much did you feel that the Telegram channel was able to alleviate some of your doubts & questions about General Surgery? | No help  Minimal help  Neutral  Somewhat helpful  Very helpful |
|  | Which mediums do you now find most useful for preparation & study of General Surgery? | Select multiple: Textbooks  Senior’s Notes  UpToDate  Online Websites  Clinics  Tutorials  Operating Theatre  Discussion with Friends, Seniors & Tutors  Web-based discussions (Telegram, Discord, Teams…) |
|  | In particular, which subspecialties do you feel you have gained the most knowledge in from the channel? Rank from most gain to least gain | Rank (from most benefit to least benefit)  Hepatobiliary  Colorectal  Endocrine  Head & Neck  Urology  Trauma/Critical Care  Breast  Vascular  Upper GI |
|  | How have your study patterns for General Surgery changed after participating in this telegram channel? Please select the mediums that you have increased in usage after participation in the channel | Choose multiple: Textbooks  Senior’s Notes  UpToDate  Online Websites  Clinics  Tutorials  Operating Theatre  Discussion with Friends, Seniors & Tutors |
|  | How suitable do you think Telegram, as a platform, is in supporting online education? | Numerical scale: 1-10 |
| **Outcome Evaluation** | | |
|  | How would you compare the effectiveness of discussions on Telegram to a traditional face-to-face tutorial? | Numerical scale, 5 as effective (0-10) |
|  | Would you like to see this implemented in the curriculum to facilitate learning when the pandemic era is over? | Numerical scale 1-10 |
|  | Would you be interested in taking on the role as a tutor for your juniors in subsequent batches | Numerical scale 1-10 |
|  | How do you feel this could have been improved? | Free text |
|  | Do you feel tutor input was sufficient? | Numerical scale 1-10 |
|  | How many discussions/questions do you participate in actively per week |  |
|  | How many discussions/questions do you passively review per week |  |
|  | Do you feel the breath/coverage of content on the channel was adequate? | Numerical scale 1-10 |
|  | Do you feel the depth of content coverage/discussions on the channel were adequate | Numerical scale 1-10 |
|  | Would you recommend this to your juniors? | Numerical scale 1-10 |
|  | What other functionalities of Telegram (bots, polls), that you are aware of, do you feel will be useful for medical education? |  |
|  | Do you feel Telegram can help to augment your medical education? | Numerical scale: 1-10 |
|  | If yes, how so? |  |
|  | How helpful do you feel anonymity would aid in responses in such a setting? | Numerical scale: 1-10 |
|  | Do you believe that more inter-participant interaction/discussions would be beneficial for your learning? | Numerical scale: 1-10 |
|  | Do you see the Telegram channel as a safe space for learning with your cohort mates? | Numerical scale 1-10 |
|  | How important do you think active participation is for your learning | Numerical scale 1-10 |
|  | How effective do you think passive participation in the discussion is for your learning? | Numerical scale 1-10 |
